# Bovine Aortic Arch, A High-Risk Variant

**DOI:** 10.7759/cureus.25456

**Published:** 2022-05-29

**Authors:** Mohammed Shaban, Pravash Budhathoki, Somin Lee, Tanushree Bhatt, Miguel A Rodriguez Guerra, May Zaw

**Affiliations:** 1 Internal Medicine, BronxCare Hospital Center, Icahn School of Medicine at Mt. Sinai, New York, USA; 2 Medicine, Montefiore Medical Center, Albert Einstein College of Medicine, New York, USA

**Keywords:** hepatic cysts, left subclavian artery, innominate artery, bovine aortic arch, aortic arch

## Abstract

The bovine aortic arch is a vascular variant related to an increased incidence of vascular and neurological complications. It should be ruled out in patients with vague neurological symptoms without a clear etiology. Our case is of a 72-year-old female patient who presented with a syncopal episode; the workup incidentally showed the aortic arch bovine variant with evidence of ischemic white matter disease more than expected for age. After reviewing the related literature, we suggest that this aortic variant is likely an independent risk factor for multiple vascular complications. A close follow-up is essential, and screening should be considered for symptomatic family members.

## Introduction

The human aortic arch (AA) branching pattern typically consists of three great vessels. It comprises the brachiocephalic trunk, left common carotid artery, and left subclavian artery [1]. This standard AA branching pattern occurs in 74% to 89.4% of adult patients. Bovine aortic arch (BAA) is the second typical AA branching pattern with an incidence of 7.2% to 21% [2].

The BAA has a different aortic arch pattern than a cattle group of animals, unlike the name bovine. In cattle, a single great innominate artery originates from the arch and is divided into three branches of the right and left subclavian arteries and carotid trunk. However, in humans, the bovine aortic arch has two great vessels from the aortic arch, the innominate artery, and the left subclavian artery. The human bovine aortic arch has two types: either the innominate artery giving rise to the left common carotid artery, or the innominate and left common carotid artery sharing a common origin from the aortic arch [1]. The bovine aortic arch is considered a normal variant of the aortic arch. Still, some literature reports that BAA can be associated with an increased risk of embolic stroke, prevalent thoracic aortic arch abnormalities, thoracic aneurysm, and dilation, and a higher mortality rate in aortic dissection [3-6]. The etiology of these complications is most probably related to the common origin of the head irrigation, the innominate artery, as the bovine aortic arch group reports prevalent aorto-thoracic vascular and neurological complications [4-6]. This is a case of a patient with a bovine aortic arch who presented with a single syncopal episode.

## Case presentation

**Figure 1:**
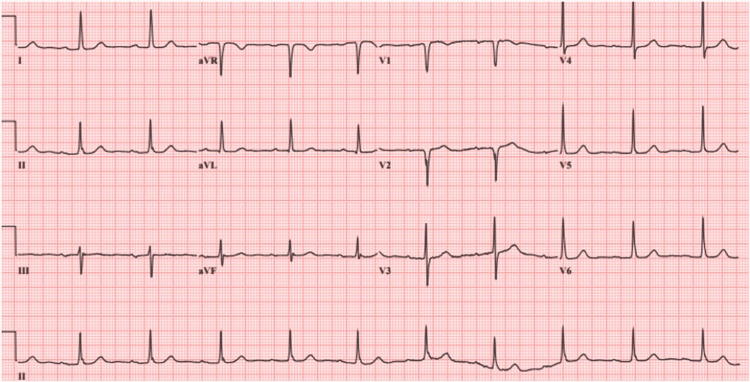
EKG on admission: Normal sinus rhythm with a first-degree AV block.

**Figure 2:**
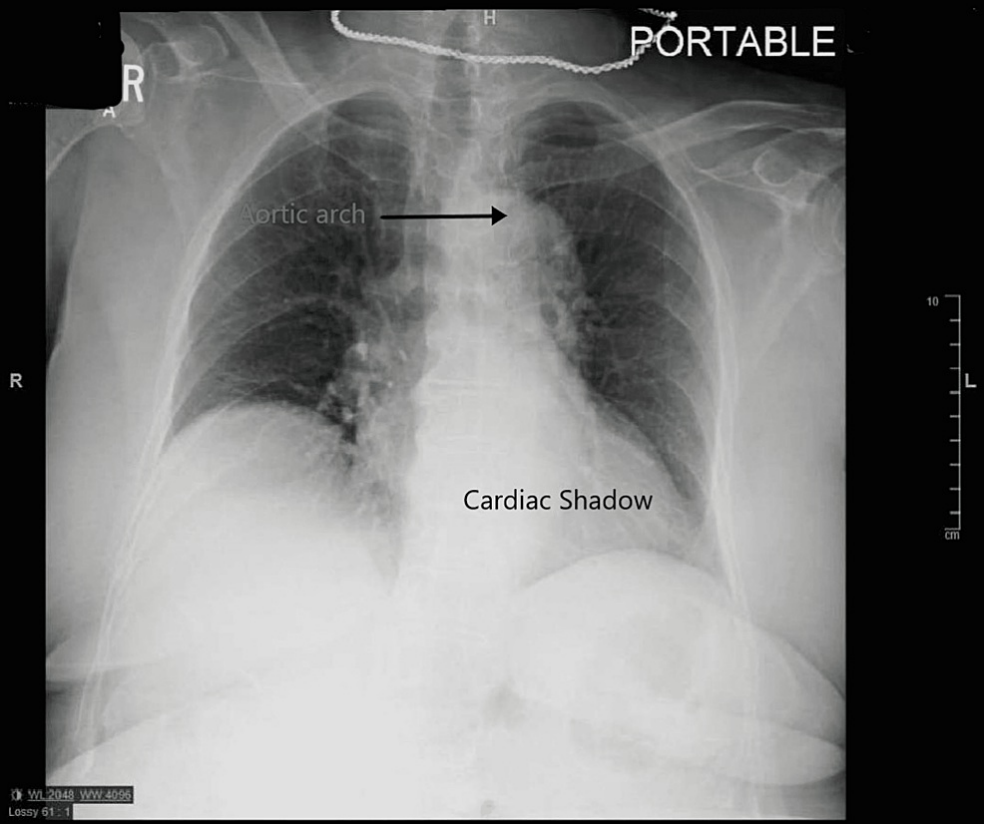
Chest radiograph showed a borderline enlarged heart with the tortuous and calcified aorta.

**Figure 3:**
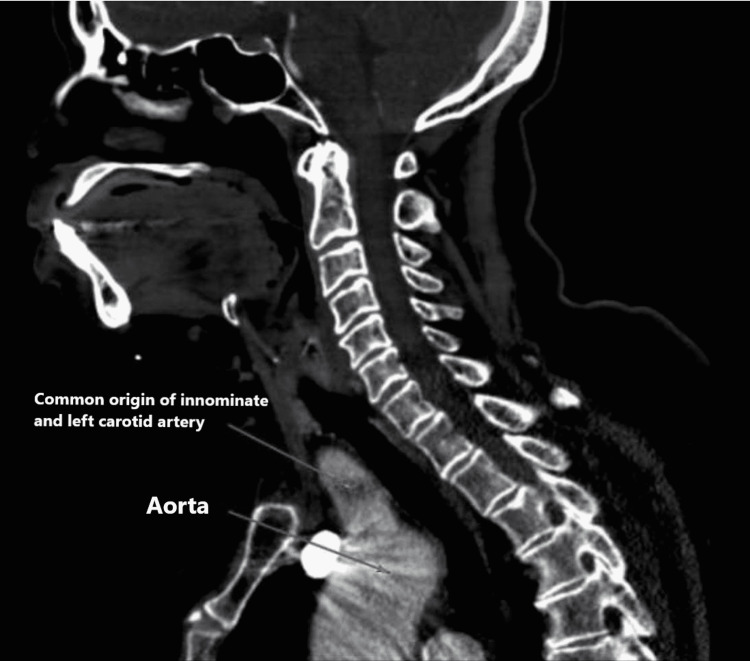
Sagittal view of CT chest shows the common origin of innominate and left carotid arteries.

**Figure 4:**
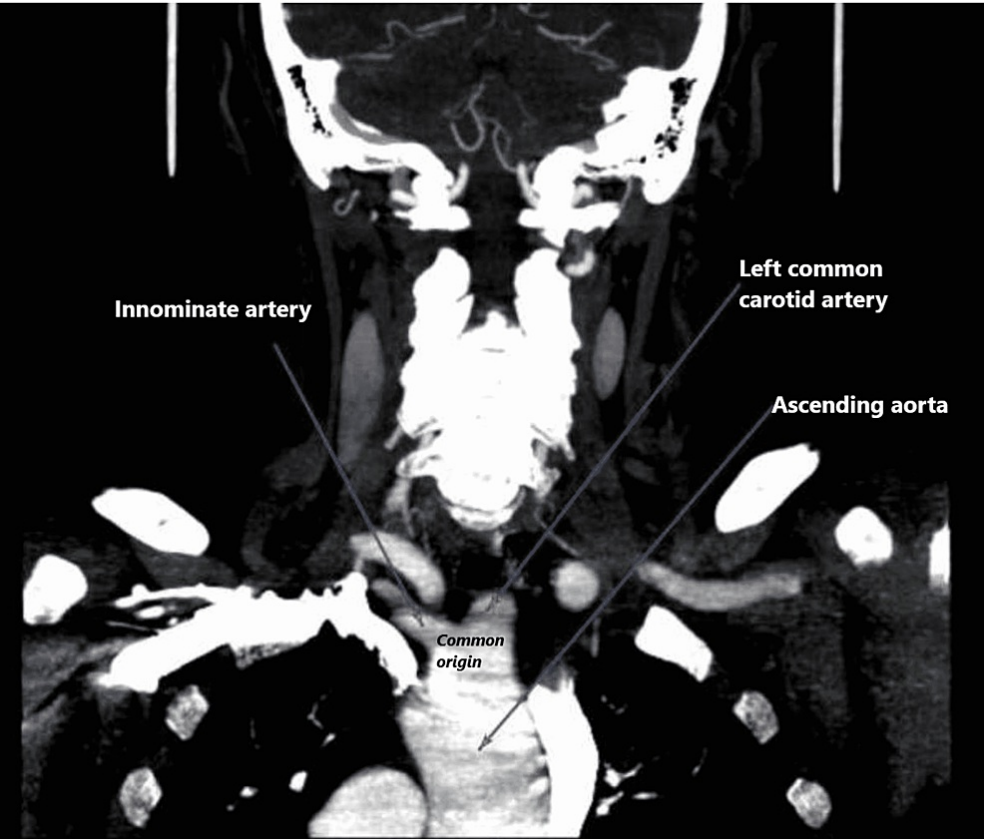
CT angiographic of the neck: Coronal view shows the bovine arch.

**Figure 5:**
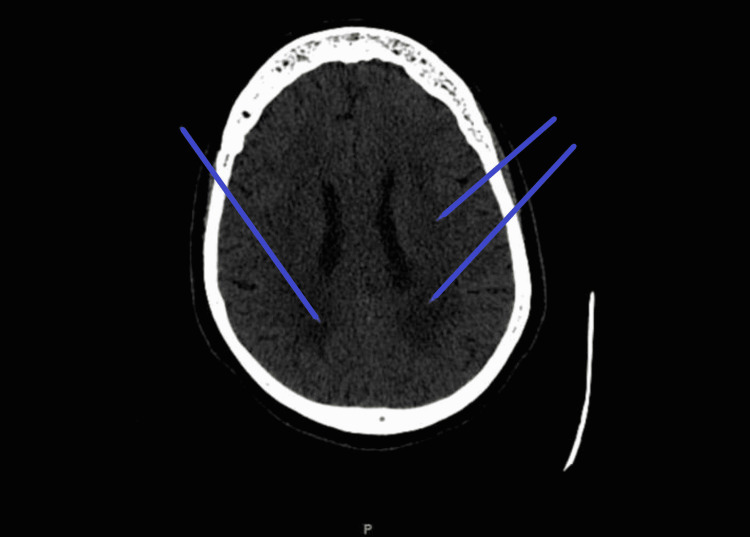
CT brain non-contrast: Transversal view showing greater than typical for age ischemic white matter disease.

**Figure 6:**
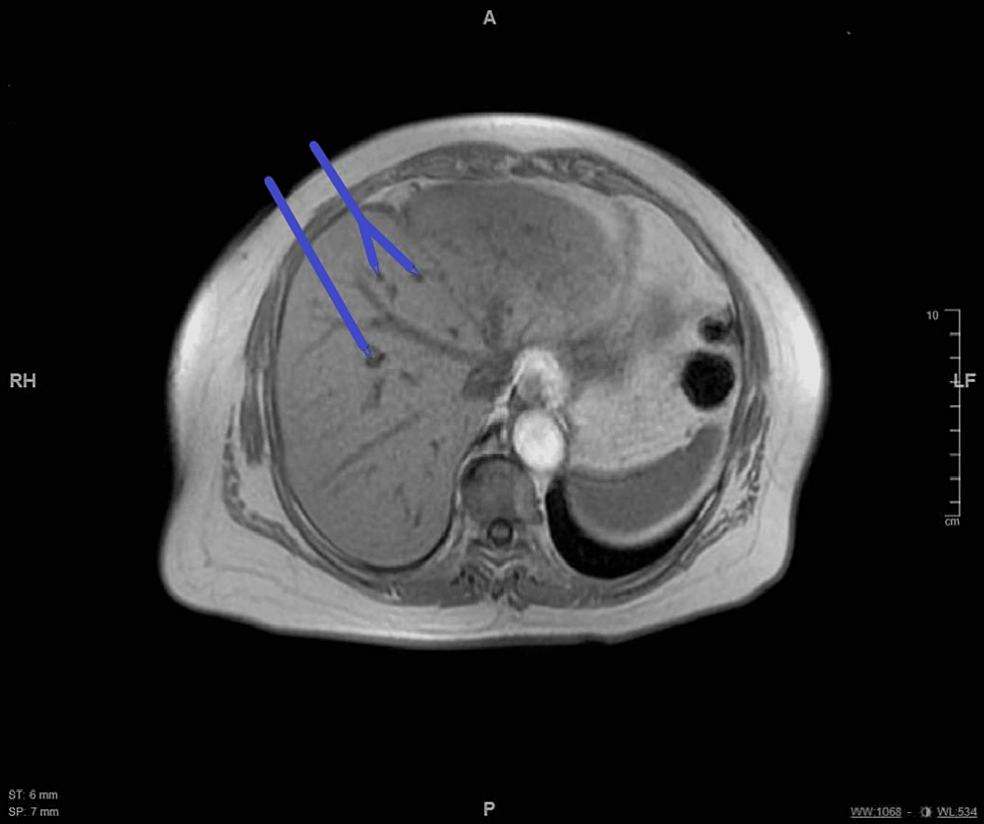
MRI: Arrows indicate the multiple hepatic cysts reported.

This is a case of a 72-year-old caucasian female who was admitted to the medical ward due to syncope. The episode lasted for a single minute and was not associated with any prodromal symptoms but was accompanied by an episode of non-bilious, non-bloody vomiting. No abnormal movements, tongue laceration, or urinary incontinence were observed. She had a medical history of hypertension, hyperlipidemia, pernicious anemia, thyroid nodules, and venous insufficiency of the right leg. She denies smoking cigarettes, illicit drugs, or alcohol use for the past fifteen years. Her vital signs, including orthostatic, were unremarkable. The physical examination was positive for a pulsatile mass on the right neck. The initial laboratories, including prolactin and creatine phosphokinase, were normal except for a mild increase in troponin T, which downtrend with subsequent measurements was likely representing demand ischemia (Table 1). The EKG on admission showed normal sinus rhythm and a first-degree AV block (Figure 1). On admission, a chest radiograph showed a borderline enlarged heart with a tortuous and calcified aorta (Figure 2). The transthoracic echocardiogram (TTE) showed normal left ventricular systolic function and concentric left ventricular hypertrophy. The computed tomographic angiographic scan of the neck revealed a bovine arch with no hemodynamically significant right or left carotid and vertebral system stenosis (Figure 3, 4); the computed tomographic of the brain revealed ischemic white matter disease greater than typical for age (Figure 5). Further chart review was remarkable for an MRI abdomen with multiple bilateral renal cysts and multiple non-enhancing simple cysts scattered in the liver, the largest measuring 1.9 cm (Figure 6). The patient was managed conservatively on the telemetry floor, including aspirin, pravastatin, and blood pressure control with losartan and nifedipine. The hospital course admission was not remarkable for any recurrence of symptoms, subclinical arrhythmias, or clinical events. The patient was discharged with uneventful outpatient follow-up over the next six months.

## Discussion

The BAA is the genetic variation in which the left common carotid artery (LCCA) originates from the brachiocephalic trunk (BT) rather than from AA. It is the regular AA branching pattern in canines, felines, or Macaque monkeys; however, it is not present in ruminant animals, such as cattle and buffalo. In another statement, BAA is a widely used misnomer [7]. Popieluszko et al.have conducted a metanalysis describing the incidence of AA variations in adults [8]. Out of 23,882 patients, classic brachiocephalic trunk, an LCCA, and a left subclavian artery counted for 80%, while BAA was 13.6%. BAA has a higher prevalence of 26.8% in African-American populations [8]. In Popieluszko's metanalysis [8], most cases were asymptomatic, but some have increased the incidence of hemorrhagic or ischemic complications during thoracic surgeries. [8] 

There are studies showing that people with bovine aortic arch (BAA) are more likely to develop ascending aortic aneurysm (AAA)than the general population [9]. In the histological evaluation for BAA compared with AAA and bicuspid aortic valve (BAV), it was shown that similar wall stiffness was found in both BAA and AAA [10] compared to BAV, which showed more microscopic evidence of elastin fragmentation, medial cystic necrosis, and mucoid ground substances accumulation. Both were less stiff and thicker than BAV aortic tissue. The increased stiffness in BAV in all conditions was related to decreased amount of elastin. However, all three subgroups have almost the same risk for aortic rupture and dissection. [9]

The BAA, especially subtype B, was significantly associated with embolic cerebrovascular accidents (CVA); it was most probably related to hemodynamic variations. People with BAA were shown to have increased shear stress resulting in inflow alterations, ultimately causing local thrombus formation predisposing to stroke. The increased shear stress could be explained by an altered angle of branching of the great vessels, particularly in type B BAA; this stress lesion is more common in the carotid and vertebral arteries rather than upper extremities vessels, which is the reason why the bovine variation has been related embolic strokes of undetermined origin in some cases [11]. Furthermore, BAA was associated with adverse neurological outcomes after carotid artery stenting procedures and thrombectomies as CVA therapeutic modalities [3]. Identifying the aortic arch variant group, including the bovine aortic arch, is vital in endovascular procedures.

The aortic arch variant, including the bovine arch itself, is a risk factor for many technical failures and neurological complications [12]. These complications include minor stroke [13], new neurological deficit [3], major stroke, and transient ischemic attack in the arch variant group, which showed 20% vs. 5.3% [12]. In a study of 833 samples for carotid stenting, neurological complications incidents reported were 10.3% in the bovine arch group observed and 4.1% in the non-bovine arch group [14]. In our case, white matter ischemia, disproportionate to age, and decreased brain volume suggest recurrent, maybe even subclinical thrombi or microthrombi events traveling to the brain. These events might be associated with the current syncopal attack our patient was experiencing. 

Interestingly, Ziganshin et al. have shown an increased association between simple renal cysts and thoracic aortic disease (TAD) that could be associated with anomalies of the aortic arch vessels (such as the bovine aortic arch and isolated left vertebral artery), equally found in males and females, contrary to the general population in which there is a male predominance [15]. They emphasized the increased simple renal cysts prevalence in aortic arch diseases up to the suggestion for potential use as a marker for early detection of aortic diseases [15]. In our case, there were numerous hepatic, renal, and pancreatic cysts supporting the possibility of a more comprehensive connective tissue pathology. Furthermore, our patient’s record of the follow-up imaging between 2018 and 2021 was not noticeable for any gross changes in these hepatic, renal, and pancreatic cysts suggesting benign pathology.

Matakas et al. [16] studied 191 cases with acute cardioembolic strokes associated with BAA. They found that the side of hemispheric involvement by imaging modalities was the left in 51.3% and right in 35.6% of the patients, with a p-value of 0.018. More severe cases had bilateral involvement in 13.1% of the studied patients. They also found a significant increase in the African race subgroup compared to the other subgroups [16]. In our Caucasian patient, despite no clinical evidence of acute motor or sensory deficit in the current or past presentations, CT without contrast showed bilateral brain volume loss associated with deep white matter infarction in the right, non-dominant, periarterial region.

Although the association of the multiple hepatic and renal cysts with the aortic arch variant is not related, our patient is an interesting case where a cerebral volume loss was present with evidence of multiple embolic events on imagining, coursing without major complication except a single syncopal episode without prodromal symptoms or further recurrence of symptoms for the following six months.

## Conclusions

The bovine aortic arch is a normal variant considered an independent risk factor for multiple vascular complications, including cerebrovascular accidents and neurological complications after endovascular procedures. This condition must be ruled out in patients with neurological symptoms without a clear etiology. Once this condition is recognized, follow-up and imaging surveillance are imperative. The patients need to be educated and constantly reinforced, primarily due to their potential complications when endovascular procedures are required. The prompt assessment and early recognition would impact the outcome of these patients and the approach for their following care and potential screening of symptomatic family members.
